# Determining essential priorities for future investigation - A national consensus exercise in Interventional Oncology (DEFINE-IO) – An a priori protocol

**DOI:** 10.1371/journal.pone.0337255

**Published:** 2026-09-18

**Authors:** Vinson Wai-Shun Chan, Blair Graham, Helen Hoi-Lam Ng, Scott Griffiths, Deevia Kotecha, Hanif Ismail, Paul Jenkins, Jim Zhong, Raghuram Lakshminarayan, Tze Min Wah

**Affiliations:** 1 UK National Interventional Radiology Research Collaborative, United Kingdom; 2 Department of Diagnostic and Interventional Radiology, St. James’s University Hospital, Leeds Teaching Hospitals NHS Trust, Leeds, United Kingdom; 3 Leeds Institute of Medical Research, Faculty of Medicine and Health, University of Leeds, Leeds, United Kingdom; 4 Department of Diagnostic and Interventional Radiology, University Hospitals Plymouth NHS Trust, Plymouth, Devon, United Kingdom; 5 Department of Vascular Radiology, Hull Teaching University Teaching Hospitals NHS Trust, Hull, United Kingdom; 6 Department of Diagnostic and Interventional Radiology, Lancashire Teaching Hospitals NHS Trust, Lancashire, United Kingdom; 7 Research and Innovation Centre, Leeds Teaching Hospitals NHS Trust, Leeds, United Kingdom; PLOS: Public Library of Science, UNITED KINGDOM OF GREAT BRITAIN AND NORTHERN IRELAND

## Abstract

**Introduction:**

Interventional Oncology (IO) is a rapidly developing subspecialty within interventional radiology (IR), offering minimally invasive, precise and targeted cancer therapies. Despite its transformative potential, IO faces challenges in identifying critical research priorities amidst limited funding and resources. The DEFINE-IO study aims to identify the most pressing research priorities in IO to guide researchers, policy makers, and funding bodies in driving innovation and improving cancer outcomes.

**Methods and analysis:**

This study comprises two phases: a modified e-Delphi process and a multi-criteria decision analysis (MCDA). The e-Delphi process will involve three rounds, with round 1 dedicated to generating research topics, followed by rounds 2 and 3, which will focus on refining and achieving consensus on the identified topics. The final consensus will yield a list of 25 research priorities, which will be evaluated during an in-person MCDA session. These priorities will be assessed against three predefined criteria: urgency, feasibility/equipoise, and affordability, to establish a strategic roadmap for research in IO.

**Ethics and dissemination:**

Formal ethics approval was deemed not necessary. Findings of the study will be presented at major conferences and published in a peer-reviewed journal. The findings will also be shared with funding bodies and policymakers to help guide future research directions.

## 1. Introduction

Interventional Oncology (IO) is now established as the fourth pillar of modern cancer care, offering a range of innovative, minimally invasive therapies [1]. Interventional oncologists utilise various techniques, broadly categorised into vascular and non-vascular approaches, to deliver precise, minimally invasive cancer therapies. Vascular IO leverages traditional vascular IR approaches via blood vessels in the body to perform procedures such as transarterial chemoembolisation (TACE) [2] and selective internal radiation therapy (SIRT) [3]. These approaches are commonly used in the management of cancers such as hepatocellular carcinoma (HCC). Non-vascular IO performs a wide range of diagnostic, curative and palliative procedures, including biopsies [4], and image-guided ablation of thyroid [5], lung [6], liver [7], kidney [8] soft tissue [9] and bone malignancies [10].

Despite its transformative potential, research in the field often proceeds without a unified strategic direction [11]. In a climate of limited research funding, there is a significant risk of fragmented efforts and the misallocation of precious resources towards questions that are not considered a priority by the clinical or patient community.

There is currently no formal consensus on the most critical research questions for IO in the UK. To ensure that future research in IO aligns with clinical needs and maximises its impact, it is essential to establish a clear framework of priorities. Therefore, the DEFINE-IO study is exceptionally timely as it directly addresses this critical gap. By applying a rigorous, evidence-based methodology utilising a modified Delphi technique [12] and the MCDA [13–15], it will build the first national research strategy for the discipline.

Therefore, this study aims to identity the most pressing research priorities in IO to provide a critical roadmap to guide researchers, policymakers, and funding bodies in directing resources towards the most pressing clinical and scientific questions in IO.

## 2. Materials and methods

### 2.1. Aims and objectives

The study’s main objective is to use a modified e-Delphi method to identify and prioritise research topics forIO. The secondary objective is to consider and reprioritise topics based on real-world deliverability.

### 2.2. Study design

This is a two-phase study integrating a modified e-Delphi method and a MCDA:

**Phase 1**: e-Delphi study to generate and reach consensus on important research topics.

**Phase 2**: MCDA to prioritise the agreed-upon research topics based on perceived urgency, feasibility/equipoise, and affordability implications.

#### 2.2.1. Rationale for modified e-Delphi method.

The Delphi method is a well established, systematic process for achieving expert consensus and has been used for research prioritisation in radiology [16], surgical oncology [17,18], and emergency care [19]. It involves multiple rounds of anonymous questionnaires with controlled feedback, promoting inclusivity and reducing bias by minimising the influence of dominant voices. Anonymity encourages honest responses, allowing participants to refine their views based on group feedback. Consensus is reached after 3 rounds.

In this DEFINE-IO study, a modified e-Delphi method will be adopted to identify research priorities, but real-world deliverability also depends on factors like urgency, feasibility/equipoise, and affordability. To address this, secondary analysis using MCDA will adjust the priorities to highlight those that are most impactful and achievable.

#### 2.2.2. *Rationale for the MCDA.*

Following the modified e-Delphi assessment, MCDA will allow study participants to assess the deliverability of each research priority against defined criteria [14,15]. Proposed criteria include the urgency of addressing the priority, the feasibility of tackling the priority, and the associated cost and resource requirements. By applying weighted values to each criterion, it will be possible to adjust and rank the priorities for implementation in a practical, real-world context [20]. While utilised in other contexts, utilisation of the MCDA is novel to research prioritisation and interventional radiology [13].

### 2.3. Scope of study

Research topics aligned with the European Curriculum and Syllabus for Interventional Oncology published by the Cardiovascular and Interventional Radiological Society of Europe (CIRSE) [21] will be included in the prioritisation exercise. Participants will be requested to suggest relevant research topics focusing on both technical and non-technical aspects of the delivery of IO.

### 2.4. Participants

#### 2.4.1. Population.

Senior trainees, specialist doctors, and consultants with a substantive post in Interventional Radiology. Participation from the wider research team such as research nurses or radiographers are also encouraged.

To be included, participants must be at the level of specialty trainee year 5 (ST5) and above and be willing to participate in all study phases or be a research nurse or radiographer in IO.

Radiologists without a substantive IR commitment, and/or unable to commit to the study timeline will be excluded.

#### 2.4.2. Conflicts of interest.

To ensure transparency and avoid potential bias, all participants will be required to declare any potential conflicts of interest (COI) in advance. This includes any paid expenses or honoraria received from any relevant companies in the 12 months prior to the study. Recognising the limited pool of experts in this specialised field and the critical need to secure a sufficient and representative sample, participants declaring COIs will not be automatically excluded. Excluding these experts could introduce significant selection bias and compromise the validity and generalisability of the study’s findings.

Instead, an active, multi-layered strategy will be adopted. First, all voting rounds will be strictly anonymised via a secure online platform, ensuring panelists remain blinded to each other’s identities and commercial ties, which naturally dilutes industry advocacy or peer alignment. Second, the initial consensus items will be drafted exclusively by the non-conflicted steering committee to prevent individual stakeholders from shaping phrasing of the item. Third, if a participant holds a direct financial interest (such as a patent) tied to a specific statement, they will be dynamically recused from voting on that item alone. Finally, the study oversight committee will independently audit raw voting data after each round to monitor for systematic bias, and a formal sensitivity analysis will be performed on the final results by recalculating the consensus matrix with conflicted responses excluded, with any substantive variations explicitly reported as a limitation.

#### 2.4.3. Incentives.

Each participant will be provided with a certificate of participation upon completion of the final study questionnaire. PubMed indexed collaborator authorship will be offered to participants completing all three rounds of the modified e-Delphi study. No monetary incentive will be provided.

### 2.5. Sampling strategy

A core participant group will be purposively recruited, aiming to ensure trainee and consultant representation from across 10 NHS regions and the three devolved nations. This includes [1] East of England [2], London [3], Midlands [4], Northeast and Yorkshire [5], Northwest [6], Southeast, and [7] Southwest [8], Wales [9], Scotland, and [10] Northern Ireland. It is anticipated that each region will contribute one IR trainee and one specialty doctor/consultant, yielding a minimum study sample of 20 participants.

Additional invites will be distributed via email and social media, using the study investigators’ existing networks. This includes the UK National Interventional Radiology Collaborative (UNITE), the British Society of Interventional Radiology (BSIR), and the Royal College of Radiologists (RCR). Invitations will promote involvement from women and groups traditionally under-represented within research.

The proposed sample size is consistent with recommended practice for Delphi studies, where panel size is determined by the need for informed judgement rather than statistical power. Previous methodological guidance suggests that relatively small, well-defined panels are sufficient to achieve stable consensus, particularly when participants are selected for their domain-specific expertise [22]. In this study, participants are purposively sampled to ensured representation across geographical regions and levels of clinical seniority, thereby capturing variation in practice while maintaining a manageable and engaged panel.

Recruitment will be stopped early once 60 participants are recruited.

### 2.6. Oversight Committee

A committee of study investigators and external representatives from UNITE, BSIR, and the RCR will oversee all stages of the study, including design, ethical considerations, questionnaire development, data analysis, and the dissemination of findings.

### 2.7. Study Process and procedures

#### 2.7.1. Study timeline.

It is anticipated that data collection will run for five months from 1^st^ August 2025–7^th^ January 2026, as detailed in Table 1.

**Table 1:** Data collection timeline.

Phase 1: e-Delphi Study		
Round 1		Date
Participant Completion	9 weeks	Commenced August 2025
Data Analysis + Report	1 week	Commence November 2025
**Round 2**		
Participant Completion	3 weeks	Commence October 2025
Data Analysis + Report	1 week	To be completed November 2025
**Round 3 and Phase 2 MCDA**		
Participant Completion (Hybrid Meeting with option to complete offline)	4 weeks	To be completed January 2026
Data Analysis + Report	2 weeks	To be completed January 2026

#### 2.7.2. Survey Platform.

A dedicated platform will be purpose built, with Microsoft forms and JISC Online Survey (https://onlinesurveys.jisc.ac.uk/) integrated for the e-Delphi study and the MCDA. This enables real time analysis of the e-Delphi results and implementation of the MCDA.

#### 2.7.3. Phase 1: e-Delphi.

This study utilises a modified Delphi approach with a fixed structure of three rounds, rather than an open-ended iteration process. A pre-specified, three-round ceiling was selected a priori to balance the need for consensus refinement with the practicalities of panel retention and alert fatigue among highly specialised clinicians. Consequently, the stopping criteria are defined as either: [1] the completion of all three sequential rounds, or [2] the achievement of stable consensus across all items prior to Round 3, at which point further rounds would be rendered redundant.

The three-round modified e-Delphi study is proposed consisting of topic generation, topic refinement and final consensus. For round 1, participants will each be invited to list up to 20 research topics or questions that they deem important for IO in the UK. To minimise ambiguity and ensure consistency in scope, participants are provided with a direct link to the European Curriculum and Syllabus for Interventional Oncology to ensure that the research topics are within scope of IO.

Topics that focus predominantly on systemic therapies (e.g., chemotherapy, immunotherapy), surgical interventions, or radiotherapy without a clear IO component will be excluded. Where topics involve multidisciplinary care, inclusion will be limited to aspects where IO plays a central or integral role (e.g., combination therapies involving ablation plus systemic treatment). Exclusion criteria for research topics will therefore include: [1] topics primarily concerning other oncological specialties without a substantive IO component; and [2] topics lacking sufficient specificity to be interpreted as a researchable question.

Submitted topics will be consolidated by the study team using qualitative thematic content analysis, with duplicate or overlapping items merged. All submissions will be screened to remove duplicates and non-relevant items based on the exclusion criteria. Ambiguous submissions will be reviewed by the study team using predefined criteria to determine whether IO constitutes the primary focus of the research question.

The resulting list of consolidated topics will form the round 2 questionnaire.

Round 2 will focus on topic refinement. Participants will be requested to assess the importance of each of the included topics against a forced-choice 9-point agreement scale, ranging from 1 (Extremely low priority) to 9 (Extremely high priority), outlined in Table 2 [17]. The median priority assigned to each topic by the participants will provide a measure of central tendency. A group median score of 1–3 indicates low priority, 4–6 indicates medium/ uncertain priority, 7–9 indicates high priority. Consensus will be determined using the IPRAS (Interpercentile Range Adjusted for Symmetry) as per the RAND/UCLA Appropriateness Method [23], with classical method as a sensitivity analysis [15]. Criteria for consensus and progression between rounds will be prespecified as follows:

**Table 2:** Forced-choice 9-point agreement scale; adapted from Schneider et al.[17].

1	2	3	4	5	6	7	8	9
Extremely Low Priority	Very Low Priority	Low Priority	Somewhat Low Priority	Medium priority	Somewhat High Priority	High Priority	Very High Priority	Extremely High Priority

The e-Delphi study will provide a prioritised list of topics, ordered by the median rating and degree of consensus. The top 25 priorities that have achieved consensus, ranked by median and interpercentile range (IPR), will be subjected to analysis using MCDA.

- High-priority consensus: median score 7–9 with agreement (IPRAS criteria met) → retained for final prioritisation.
- Low-priority or medium-priority consensus: median score 1–6 with agreement → excluded from further rounds.
- No consensus: Any median without agreement → carried forward to Round 3.

Findings from this round will be compiled into a feedback report, where each participant will see 1) their own rating, 2) the group median score, 3) distribution of responses and 4) consensus classification for each topic. This report will be used to facilitate round 3.

Round 3 will determine final consensus. Participants will be asked to repeat an identical questionnaire (re-rate topics) to that used during round 2, but with answers informed by the feedback report to inform their choices. Repeat analysis of this data, using identical methodology to that employed following round 2, will determine final priorities and consensus. Analysis will follow the same methodology as Round 2. Only topics classified as uncertain or lacking consensus in Round 2 will be prioritised for inclusion in Round 3 if questionnaire burden is excessive. Final prioritisation will be based on Round 3 median scores and consensus status.

In rounds 2 and 3, the participants will be asked to rate their expertise for each statement on a scale of no expertise, low expertise, moderate expertise, or high expertise. These data will be used to perform sensitivity analyses to assess whether prioritisation differs when weighting responses by self-reported expertise..

#### 2.7.4. Phase 2: MCDA.

An in-person meeting with a hybrid option via Microsoft Teams will be planned to facilitate the MCDA process. This method provides a structured framework for making complex decisions by systematically evaluating various options against multiple, often competing, criteria. Reprioritising topics based on MCDA scores aims to augment e-Delphi findings by giving an indication of the real-world deliverability of each research topic. To ensure geographic accessibility and mitigate nationwide logistical challenges, the session is operationally structured as a hybrid framework from the outset. A unified MCDA form will be utilised to collect all performance scores and preference weights in real time, ensuring the data collection methodology functions identically for both physically present and remote delegates. The MCDA will be conducted in a facilitated workshop setting to ensure all participants have a shared understanding of the process and to manage group dynamics. Participants from the e-Delphi phase will be invited to take part. The MCDA process will consist of three main stages: problem structuring, performance scoring and preference elicitation (weighting). As a definitive contingency plan, if full in-person gathering becomes unfeasible due to travel disruption or institutional restrictions, the framework allows an immediate pivot to a fully virtual, synchronized video-conferencing format using the identical digital voting infrastructure, safeguarding the study timeline and data integrity.

2.7.4.1. Problem Structuring: The decision problem is to rank the top 25 research priorities (alternatives) based on their real-world deliverability.

- Alternatives: The 25 highest-ranking research topics identified from the e-Delphi process.
- Criteria: The alternatives will be evaluated against three predefined criteria. To ensure consistent interpretation, each criterion is defined as follows:
  - Urgency: The degree to which the research topic addresses a pressing clinical or patient need that requires immediate attention. This considers the potential for the research to rapidly impact patient outcomes or resolve significant existing uncertainties in clinical practice.
  - Feasibility/Equipoise: The likelihood that a research project on this topic can be successfully designed, executed, and completed within the typical constraints of the UK research environment. This includes considerations of patient recruitment, ethical approval complexity, the availability of required technical expertise and infrastructure, and whether there is equipoise for that investigation.
  - Affordability: The estimated cost and resource requirements for conducting research on this topic. This is a ‘cost’ criterion, where a more affordable topic is considered more favourable. It encompasses not only direct financial costs but also demands on personnel, equipment, and facilities.

2.7.4.2. Performance Scoring: Participants will assess the 25 research topics against each of the three criteria, populating a performance matrix.

- Scoring method: A constructed scale with direct rating will be used. For each criterion, participants will rate each of the 25 topics using a five-point Likert Scale with clearly defined performance levels to ensure consistency. The scale will be anchored as follows:
  - Urgency: 1 (Very Low Urgency) to 5 (Very High Urgency)
  - Feasibility/Equipoise: 1 (Very Low Feasibility/Equipoise) to 5 (Very High Feasibility/Equipoise)
  - Affordability: 1 (Very High Cost/Low Affordability) to 5 (Very Low Cost/High Affordability)
- Normalisation: The raw scores from the Likert Scale will be converted to a common numerical value scale from 0 to 100, where 0 represents the worst possible performance and 100 represents the best. This normalisation allows for the meaningful aggregation of scores across different criteria.

**Fig 1:**
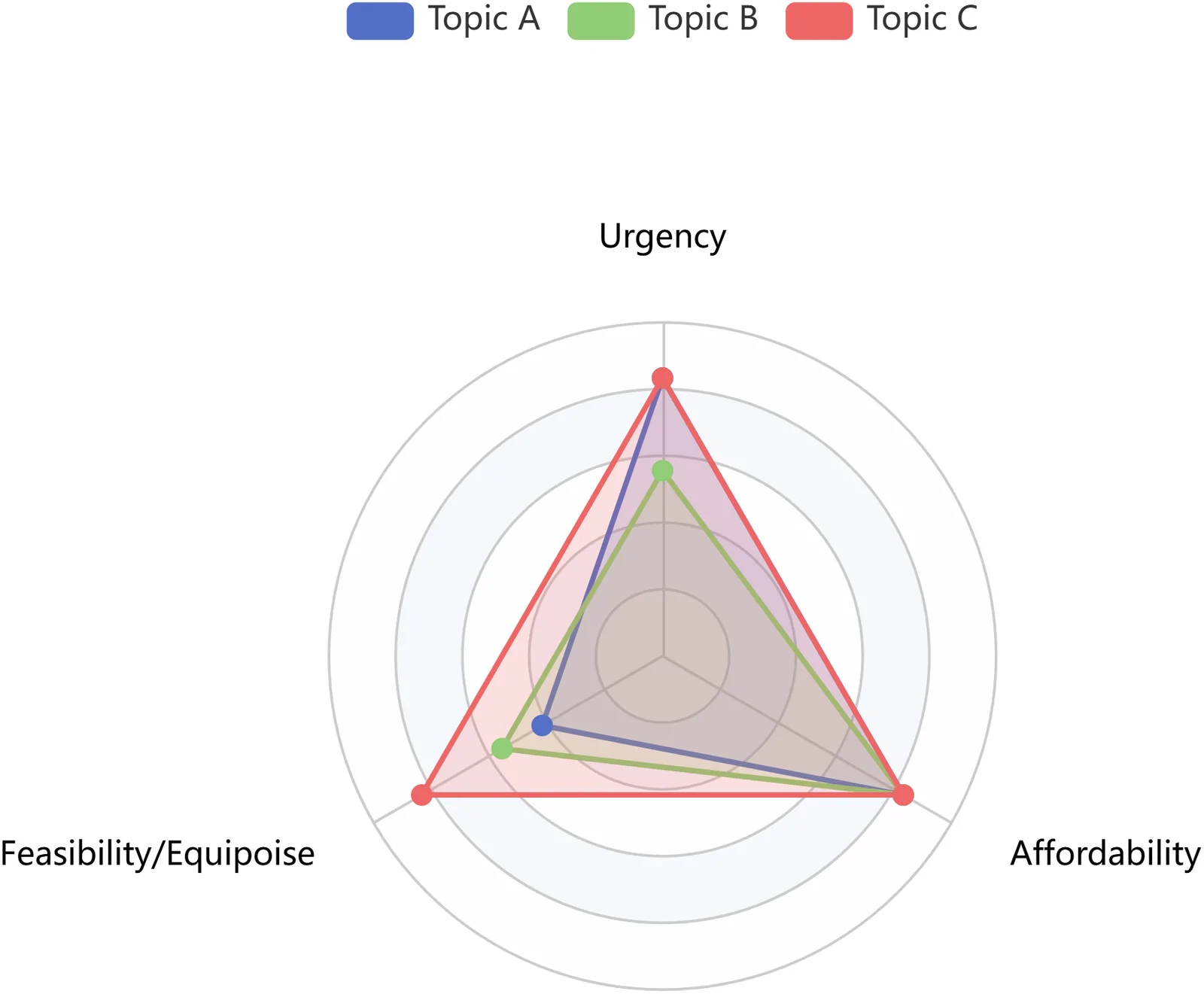
Example of how a spider chart may be used, following MCDA, to assess deliverability of topics based on urgency, feasibility/ equipoise and affordability. Whilst Topic A is most urgent, it is less feasible to investigate than Topic C. All three topics are equivalent in terms of affordability, and topic A and C are equivalent urgency.

2.7.4.3. Criteria Weighting: In the first instance, each criterion will be assigned an equal weighting (i.e., 0.33) to provide a neutral baseline for comparison. Presentation of data using spider charts will provide an intuitive means of presenting disaggregated data and may assist onward decision making and selection of topics (example—Fig 1)

To move beyond a simple assumption of equal importance, participants will elicit criteria weights that reflect their collective judgement on the relative importance of each criterion. The Swing weighing method will be used as a weighting method due to its theoretical robustness in capturing preference trade-offs [15]. This approach ensures that weighting reflects explicit value judgements rather than implicit assumptions of equal importance. Weight elicitation will be undertaken exclusively by interventional oncologists. This approach is intentional, as the primary aim of this study is to identify and prioritise research questions specifically within the field of IO, from the perspective of those who both deliver and shape IO services. Restricting weight elicitation to this group ensures that the resulting prioritisation reflects domain-specific expertise and real-world decision-making constraints inherent to IO practice. To mitigate potential bias arising from a single professional group, the study will include participants from a range of IO practice settings and levels of experience (including but not exclusive to research nurses, radiographers), and weighting will be conducted using structured and independent scoring procedures. Furthermore, sensitivity analyses will be performed based on level of experience to explore how alternative weighting assumptions may influence prioritisation outcomes. Prior to the session, a pilot process will be completed within the UNITE Collaborative. The process is as follows [15]:

1 Establishment of a Baseline Scenario: A baseline scenario describing a hypothetical research topic that performs at the lowest possible level on all three criteria will be presented to all participants. This establishes a common reference point for the weighting exercise.
2 Identification of the Primary Criterion: Participants will be asked the following question: “If you could change the performance of just one criterion from its worst level to its best level, which single change would provide the most significant improvement?”
  - Each Participants will independently write down their selected criterion.
  - A formal vote will be conducted to aggregate the individual selections. The criterion receiving the highest number of votes will be designated as the primary criterion.
3 Benchmark Scoring: The primary criterion identified in the preceding step will be assigned a benchmark score of 100 points. This score will serve as the anchor for the relative scoring of the remaining criteria.
4 Relative Scoring of Secondary Criteria: The remaining two criteria will be scored relative to the 100-point benchmark. For each criterion, participants will be asked: “Compared to the 100-point value assigned to the ‘swing’ of the primary criterion, how many points would you assign to the ‘swing’ of this criterion?”
  - Each participant will privately record a score between 0 and 100 for each of the remaining criteria.
5 Data Aggregation: The scores for each criterion from all participants will be collected. The arithmetic mean of the scores for each criterion will then be calculated to produce a single, aggregate score.
6 Normalization of Weights: The aggregate scores will be normalized to create a set of weights that sum to 1.0.

Independent scoring prior to aggregation will be used to reduce the risk of dominance bias and group conformity effects. Sensitivity analyses will be conducted to determine the effect of weighting priorities towards each of urgency, feasibility/equipoise and affordability.

### 2.8. Data Analysis

#### 2.8.1. Content analysis for topic generation.

The Delphi statements will be developed through a systematic thematic analysis of the initial, unstructured research suggestions provided by participants in round 1. To ensure a rigorous and clinically relevant structure, the analysis will be grounded in the framework of the CIRSE European Curriculum and Syllabus for Interventional Oncology [21]. Initially, all suggestions will be reviewed to identify recurring topics and concepts. These will then be mapped onto the major sections of the CIRSE curriculum, a process that will allow for the establishment of several core research themes. Each individual suggestion will then be coded to an appropriate theme. Overlapping or related ideas will subsequently be synthesized and refined into distinct, unambiguous statements formulated to be clear, concise, and suitable for rating in an e-Delphi survey. This iterative process will ensure that the full spectrum of participant ideas, from broad concepts to highly specific procedural questions, is captured and logically organised.

#### 2.8.2. Descriptive statistics.

A comprehensive set of descriptive statistics will be calculated to summarise the data from each round of the e-Delphi study and the MCDA study. This analysis will go beyond basic descriptives to provide a detailed overview of the panel’s composition, engagement and emergent consensus.

The statistical summary will include but is not limited to participant characteristics including their professional role, years’ experience, geographic location, to describe the expert panel’s composition. Furthermore, response rates and engagement metrics will also be reported to ensure panel stability and engagement over time.

Following the thematic analysis of qualitative data from round 1, a descriptive summary of the emergent research topics will be presented. This will outline the key domains of the research identified by the panel.

For the e-Delphi study, basic descriptive statistics will include participant characteristics, survey characteristics at each round (including completion and time to completion for each survey), median priority assigned to each topic, and IPRAS for each item.

For the MCDA component, basic descriptive statistics will include survey characteristics, findings using equally weighted criteria, and sensitivity analyses. Presentation of findings using spider chart(s).

#### 2.8.3. Consensus Criteria (e-Delphi).

To assess group consensus and the relative priority of each research statement from Round 2 onwards, measures of central tendency (median priority score) and dispersion will be calculated. Consensus will be determined using the RAND/UCLA method, where a statement is deemed to have reached consensus if its interpercentile range (IPR) is smaller than its Interpercentile Range Adjusted for Symmetry (IPRAS). The IPRAS will be calculated for each item using the established formula: IPRAS = 2.35 + (1.5 * AI), where AI represents the asymmetry index [23].

#### 2.8.4. Sensitivity analyses.

DEFINE-IO is a prioritisation exercise designed for the wider IO community, aiming to capture a broad and representative perspective across the field, therefore it is acknowledged that participants will naturally have differing levels of expertise in specific areas, but this diversity is an important part of reflecting the collective view of the IO community. However, it is acknowledged that some panellist may feel less confident in rating certain topics that they have no or less expertise in, hence a sensitivity analyse will be performed for the e-Delphi study with the participant’s self-perceived expertise. Furthermore, where necessary and feasible, sensitivity analysis will be performed based on the panellists’ year of experience, area of practice, and their baseline characteristics. Sensitivity analyses will also be performed in the MCDA utilising different weights.

### 2.9. Attrition Bias

To ensure absolute data integrity, all online questionnaire fields will be set as mandatory, eliminating item non-response within any active round. Methodologically, participation in subsequent e-Delphi iterations is strictly sequential; participants must complete Round 1 to be eligible for Round 2, and Round 2 to progress to Round 3. To maintain panel engagement and mitigate the risk of attrition bias within this highly specialised cohort, several proactive strategies will be employed. Regular and personalised communication will be maintained, and participants will receive summarized, anonymised feedback from the previous round to directly demonstrate the value and impact of their ongoing contribution. Surveys will be designed to be as concise as possible to respect participants’ time, supported by automated weekly reminders and direct steering committee outreach to non-responders before an active window closes. In the event of irreversible panelist drop-out between rounds, the consensus percentages for subsequent iterations will be dynamically recalculated based strictly on the active denominator of that round’s completing cohort. Furthermore, to ensure complete transparency, the demographic and professional characteristics of participants who complete all rounds versus those who withdraw will be statistically compared to formally assess and report on any systematic differences.

## 3. Ethical considerations

Formal ethical review and approval by an Institutional Review Board or Ethics Committee were deemed not necessary for the DEFINE-IO study. This decision is based on the nature of the research, which qualifies as a national consensus exercise focused on professional opinion elicitation rather than study on human subjects or clinical trial. Specifically, no human subjects research is involved. The participants are expert clinicians providing their professional judgment on future research priorities and deliverability, not contributing biological or personal data about themselves or patients. The study does not involve intervention, collection of clinical data, or testing of hypotheses related to health outcomes. According to the HRA decision tool this study does not require NHS Research Ethics Committee review.

All panellists are provided with detailed information about the study’s purpose, procedures, risks, and benefits, with electronic written consent obtained prior to participation. Confidentiality is maintained by anonymising data and securely storing it in password-protected files, accessible only to the research team. Additionally, data protection measures ensure compliance with the UK General Data Protection Regulation (GDPR) and the Data Protection Act 2018.

## 4. Dissemination of results

The study results will be compiled into a comprehensive report for participants and prepared for submission to peer-reviewed journals in interventional radiology. Findings will be presented at national conferences, such as the IOUK Annual meeting, BSIR Annual Scientific Meeting, and shared with funding bodies and policymakers to help guide future research directions.

To enhance clinical translation, the results will be actively disseminated to organisations involved in guideline development and service standard setting, including national professional societies (e.g., BSIR, IOUK). Engagement with these stakeholders will facilitate consideration of prioritised research topics in the development and updating of clinical practice guidelines and consensus statements.

In addition, the prioritised research agenda will inform future priority exercises, such as James Lind Alliance priority setting partnerships to inform the design, commissioning, and approval of future clinical studies in IO. By aligning identified priorities with funding calls and trial development pipelines, this work aims to support the initiation of high-impact, clinically relevant studies.

Where appropriate, findings will also inform optimisation of diagnostic and therapeutic strategies by highlighting areas of uncertainty, variation in practice, or unmet clinical need within IO. This may guide the development of prospective trials, registry studies, and service evaluations, thereby strengthening the evidence base underpinning IO practice.

## 5. Potential limitations

Potential limitations include attrition between e-Delphi rounds and the MCDA phase, which may impact response rates. Additionally, there is a risk of subjective bias in scoring and weighting criteria. Overrepresentation of certain regions or institutions could skew the prioritisation of results.

To mitigate these risks, efforts will be made to ensure broad national representation by recruiting panellists across multiple NHS regions and institutions. Attrition will be minimised through regular, structured communication and clear timelines for participation.

In addition, a prespecified stratified analysis will be undertaken to explore potential regional variation in responses. Consensus results and prioritisation rankings will be compared across NHS regions (e.g., by geographical grouping or devolved nations where appropriate) to assess for systematic differences. Where variation is identified, this will be reported and interpreted to provide context for the generalisability of findings. Even in the absence of significant differences, this analysis will enhance the transparency and robustness of the study.
